# Managing Nonunions and Fracture-Related Infections—A Quarter Century of Knowledge, and Still Curious: A Narrative Review

**DOI:** 10.3390/jcm14217767

**Published:** 2025-11-01

**Authors:** Jonas Armbruster, Benjamin Thomas, Dirk Stengel, Nikolai Spranger, Paul Alfred Gruetzner, Simon Hackl

**Affiliations:** 1 BG Klinik Ludwigshafen, Department for Orthopaedics and Trauma Surgery at Heidelberg University, Ludwig-Guttmann-Str. 13, 67071 Ludwigshafen, Germany; 2 BG Klinik Ludwigshafen, Department of Hand, Plastic, and Reconstructive Surgery, Burn Center at Heidelberg University, Ludwig-Guttmann-Str. 13, 67071 Ludwigshafen, Germany; 3 BG Kliniken-Klinikverbund der Gesetzlichen Unfallversicherung gGmbH, Leipziger Pl. 1, 10117 Berlin, Germany; 4 Department of Trauma and Orthopaedic Surgery, BG Klinikum Unfallkrankenhaus Berlin gGmbH, 12683 Berlin, Germany; 5 Department of Trauma Surgery, BG Unfallklinik Murnau, Professor-Küntscher-Straße 8, 82418 Murnau, Germany

**Keywords:** low-grade infections, orthoplastic surgery, bone graft substitutes, allografts, nonunion, fracture-related infections

## Abstract

Nonunions and fracture-related infections represent a significant complication in orthopedic and trauma care, with their incidence rising due to an aging, more comorbid global population and the escalating threat of multi-resistant pathogens. This narrative review highlights pivotal advancements in diagnostics and therapeutic approaches, while also providing an outlook on future directions. Diagnostic methodologies have significantly evolved from traditional cultures to sophisticated molecular techniques like metagenomic next-generation sequencing and advanced imaging. Simultaneously, therapeutic strategies have undergone substantial refinement, encompassing orthoplastic management for infected open fractures and the innovative application of antibiotic-loaded bone substitutes for local drug delivery. The effective integration of these possibilities into daily patient care critically depends on specialized centers. These institutions play an indispensable role in managing complex cases and fostering innovation. Despite considerable progress over the past 25 years, ongoing research, interdisciplinary collaboration, and a steadfast commitment to evidence-based practice remain crucial to transforming management for the future.

## 1. Introduction

Over the last decades, healthcare has shifted from empiric to evidence-based medicine, fundamentally transforming clinical decision-making and patient care [1]. While clinical practice guidelines evolved in many areas, this transition has been particularly challenging in managing nonunions and fracture-related infections (FRI), a field characterized by complex and heterogeneous patient populations, varying pathogen spectra, and diverse treatment approaches [2,3,4]. While significant scientific advancements have been made in understanding and treating these infections, some critical questions remain unanswered, leaving clinicians to navigate a landscape that still demands experience-driven intuition and rigorous scientific evaluation [5]. Musculoskeletal infections encompass a range of conditions, including fracture-related infections, osteomyelitis, and nonunions. Despite the distinct pathophysiology of fracture-related infections, osteomyelitis, and nonunions, these entities share several features: prolonged treatment courses, high rates of recurrence, and a substantial socioeconomic burden [6,7]. Over the past 25 years, significant strides have been made in refining diagnostic criteria, optimizing surgical and antimicrobial strategies, and developing novel biomaterials to improve patient outcomes.

Recent data suggest nonunions occur in 2–5% of fractures, while FRIs complicate 1–2% of closed cases but can exceed 30% in severe open injuries [8,9,10]. These rates vary globally, and a significant data shortage exists for low- and middle-income countries, which carry a disproportionately high trauma burden and risk of complications. Furthermore, individual risk factors such as smoking and diabetes significantly increase a patient’s susceptibility to poor healing outcomes. One of the most challenging aspects is the early and accurate diagnosis of infection. Laboratory markers such as C-reactive protein (CRP) and white blood cell count have long been used as indicators. Still, their sensitivity and specificity remain limited, particularly in patients with chronic infections or underlying inflammatory conditions [11]. Recent advances in molecular diagnostics, imaging, and biomarker research have provided promising new tools for the more precise and earlier detection of nonunions and FRIs; however, their routine clinical application is still evolving. These developments will be discussed in detail in Section 2 of this review.

Another primary research focus has been refining surgical techniques and implant-related infection prevention. Radical debridement remains the cornerstone of surgical management, as necrotic bone serves as a persistent nidus for infection, impairing the effectiveness of systemic antibiotic therapy [3,12]. The increasing implementation of orthoplastic approaches, particularly in treating infected open fractures, has significantly improved functional outcomes by integrating soft tissue and bone reconstruction in a single-stage or multi-stage concept. The principles and current best practices of this approach will be explored in Section 3.

Despite advances in surgical methods, targeted antimicrobial therapy is pivotal in infection control. Historically, antibiotic-loaded cement spacers and microbiologically tailored systemic antibiotics have formed the cornerstone of treatment. However, antibiotic-loaded bone substitutes have emerged as a promising adjunct to systemic therapy. These biomaterials provide structural support and osteoconductive benefits while achieving high local antibiotic concentrations, thereby reducing the risk of reinfection and limiting systemic toxicity [2,13]. Section 4 will analyze the latest developments in biomaterial-based antimicrobial strategies for treating FRIs or nonunions.

In addition to these advancements, the role of highly specialized centers in managing complex musculoskeletal infections has become increasingly relevant. In Germany, BG Kliniken (the Group of Hospitals of the Federal Social Accident Insurance, DGUV) play a crucial role in handling these challenging cases, serving as national referral and international collaborating centers. Their expertise and impact on patient outcomes are discussed in the final Section 5.

While the past 25 years have brought remarkable progress in understanding nonunions and fracture-related infections, the topic remains dynamic, with ongoing research continuously shaping clinical practice. This review provides an evidence-informed synthesis of these developments, prioritizing consensus definitions and guidelines, high-quality reviews, and landmark clinical studies from the last quarter-century. It summarizes key advancements, highlights persistent challenges, and explores the emerging concepts that will define the next era of managing nonunions and fracture-related infections.

## 2. Diagnostic Challenges and Advances in Nonunions and Fracture-Related Infections

Musculoskeletal infections encompass a broad spectrum of diseases, including fracture-related infections, osteomyelitis, septic arthritis as well as periprosthetic joint infections. All conditions significantly impact patient morbidity, length of hospitalization, and healthcare costs. The insidious nature of many of these infections, particularly in immunocompromised individuals (e.g., patients with diabetes mellitus) or those with indwelling metallic or composite orthopedic implants, makes early detection both critical and challenging. Inadequate or delayed diagnosis can result in persistent infection, mechanical failure of prostheses or osteosynthesis hardware, bone and soft tissue defects, and, in severe cases, limb loss or mortality.

**Figure 1:**
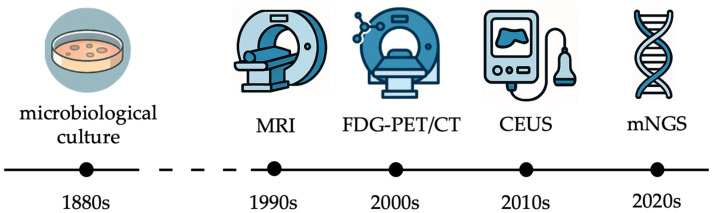
Milestones in the diagnosis of nonunions and fracture-related infections during the last half century (MRI: magnetic resonance imaging; FDG-PET/CT: fluorodeoxyglucose positron emission tomography/computed tomography; CEUS: contrast-enhanced ultrasound; mNGS: metagenomic next-generation sequencing).

Over the last decades, clinicians and researchers have made substantial progress in understanding the pathogenesis of nonunions and FRI, the role of biofilms, and immune evasion mechanisms. Concurrently, diagnostic technologies have evolved, shifting from culture-based methods to molecular assays, immunodiagnostic biomarkers, and advanced imaging (Figure 1).

### 2.1. Classical Microbiological Methods

Beginning with the work of Louis Pasteur and Robert Koch in the 1860s, microbiological culture has been the gold standard for diagnosing musculoskeletal infections [14]. While culture techniques have been integral to microbiology for over a century, their application in musculoskeletal infections faces several limitations. Many fastidious or slow-growing organisms require specific conditions and prolonged incubation periods. In the early 2000s, detection rates from synovial fluid or periprosthetic tissue cultures were highly variable, with reported sensitivity estimates ranging from 60% to 80%. Prior antibiotic exposure and suboptimal sampling techniques contributed to false-negative results [15].

The introduction of enriched blood culture bottles (e.g., BACTEC™ and BacT/ALERT™) around the mid-2000s marked a milestone. These systems enable direct inoculation of aspirated synovial fluid, thereby enhancing the yield, particularly for slow-growing organisms such as *Cutibacterium acnes*. Furthermore, Schäfer et al. emphasized the need for obtaining multiple intraoperative tissue samples—ideally five or more—to increase diagnostic accuracy and account for potential contaminants [16]. Hackl et al. have consistently argued for the importance of long-term culturing, showing that this method more than doubles the pathogen detection rate in apparently aseptic tibial shaft nonunions, from 19.3% to 42.0% [11]. The clinical significance of identifying these late-appearing pathogens was confirmed in a follow-up study on femoral and tibial nonunions. It found that negative long-term outcomes were just as prevalent in patients with late-detected pathogens as in those with positive short-term cultures, affirming that a delayed diagnosis does not imply a less severe prognosis [17].

Meanwhile, sonication of explanted prostheses, proposed since the early 2000s, has substantially enhanced the detection of biofilm-associated sessile bacteria. This technology releases adherent organisms into the surrounding fluid, which can then be cultured with higher sensitivity, particularly in patients who have received pre-operative antibiotics [18]. In 2020, the sonication method was expanded from joint arthroplasties to fracture fixation hardware, such as plates, screws, and nails, and newer methods of sonication, such as membrane-filtrated sonication, were introduced. Trenkwalder et al. showed a considerably higher polymicrobial detection rate with membrane filtration of the sonication fluid compared to tissue and sonication fluid broth culture [19,20]. However, in the context of chronic fracture-related infections, such as septic nonunion, it remains essential to establish a precise cut-off value to differentiate between bacterial contamination and infection. In a multicentric study, Trenkwalder et al. defined a cut-off value for differentiating between septic and aseptic nonunion as ≥ 13.6 CFU/10 mL sonication fluid [20].

### 2.2. Biomarker-Based Diagnostics

Inflammatory biomarkers in peripheral blood and synovial fluid play a key role in infection workup. They may assist in the selective harvesting of tissue samples and in interpreting inconclusive culture results. Conventional tests, such as C-reactive protein (CRP)—introduced in daily clinical practice around 1980—and the erythrocyte sedimentation rate (ESR), remain diagnostic workhorses due to their widespread availability and cost-effectiveness. Unfortunately, these assays have only modest diagnostic accuracy, particularly in chronic or low-grade infections [11,21]. For instance, in patients with presumed aseptic nonunion of long bones and normal CRP levels, the risk of missing low-grade infections may reach 26% [22]. In 2014, the introduction of point-of-care (POC) analysis for alpha-defensin, an antimicrobial peptide released by neutrophils, marked a milestone in the laboratory verification of periprosthetic joint infections. Results are available within ten minutes, show excellent sensitivity and specificity, and maintain performance even in patients receiving antibiotics [23]. In 2020, calprotectin, another neutrophil-derived protein, gained attention as an accurate biomarker in synovial fluid, thereby enhancing diagnostic precision when combined with synovial CRP [24]. Other promising candidates include interleukin-6 (IL-6), presepsin, and lipocalin-2. All these markers reflect systemic and localized immune responses, providing complementary data to identify infections.

Still, no single blood-based biomarker, or even a set of markers, can confirm or exclude infection of an arthroplasty or fracture fixation device (Table 1). Novel laboratory tests, ideally POC tests, are urgently required to rule infections in or out [25].

### 2.3. Molecular Diagnostics

Molecular techniques have revolutionized infection diagnostics by allowing for the rapid and precise identification of pathogens, even when conventional cultures fail. Polymerase chain reaction (PCR), initially employed for single or multiplex pathogen detection, was expanded to include broad-range 16S rRNA amplification. This approach facilitates the detection of virtually all bacterial species. Nevertheless, vigilance is required to distinguish contaminants from true pathogens [30].

The 2010s witnessed the emergence of real-time PCR (qPCR) and multiplex panels, which successfully detected pathogens in periprosthetic joint infection and spinal osteomyelitis. However, these assays are constrained by their reliance on pre-defined targets, which limits their utility when rare or unexpected pathogens are involved [31].

Metagenomic next-generation sequencing (mNGS) addressed this gap. In contrast to PCR, mNGS can directly identify bacteria, viruses, fungi, and even antimicrobial resistance genes in clinical specimens, thereby offering a hypothesis-free diagnostic approach [32]. Numerous studies have demonstrated the clinical utility of mNGS. For instance, Zhao et al. observed higher sensitivity with mNGS than culture in diagnosing osteoarticular infections, particularly among patients with prior antimicrobial exposure [33]. Ivy et al. reported mixed infections and rare anaerobes in culture-negative periprosthetic joint infection cases using mNGS [34].

Challenges associated with this technology include high costs, the need for rigorous contamination control, and the difficulty of interpreting polymicrobial results. Despite these challenges, mNGS is gaining interest and importance, particularly in cases that are complex or refractory. Yet, more and larger studies are needed to better define the diagnostic value of molecular techniques, especially in fracture-related infection [35,36].

### 2.4. Imaging Modalities

**Figure 2:**
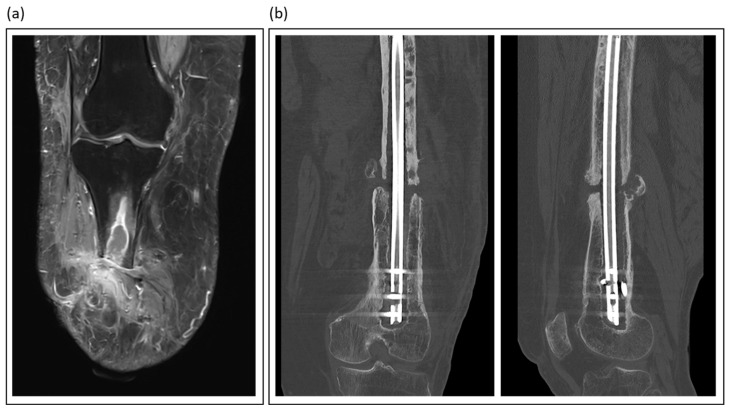
Illustrative imaging of nonunions and fracture-related infections: (**a**) T2-weighted MRI of a patient with traumatic lower leg amputation, showing an intraosseous abscess and soft tissue changes indicative of chronic infection; (**b**) Coronal and sagittal CT views of an atrophic femoral nonunion, a condition often linked to low-grade infection.

Imaging is key in diagnosing nonunions and fracture-related infections (Table 2). It confirms the presence and extent of disease, guides surgical planning, and monitors treatment response. Despite their wide availability, plain radiographs and computed tomography (CT) are of limited accuracy and value in this scenario, particularly in early stages of chronic infection [37]. However, CT is invaluable in the diagnostic process and therapeutic planning of nonunion management (Figure 2).

Magnetic resonance imaging (MRI) emerged as the gold standard for evaluating osteomyelitis and intraousseus abscess formations (Figure 2). Its excellent soft tissue contrast allows for the visualization of marrow edema, abscess formation, and sinus tracts. Advanced sequences, such as contrast-enhanced and diffusion-weighted imaging, further enhance MRI sensitivity [35]. Love et al. emphasized the unparalleled value of MRI for the early detection of septic bone lesions, particularly in challenging situations such as diabetic foot infections [38]. However, metal artifacts make it difficult to diagnose infections associated with fracture fixation devices (plates, screws, and nails).

While many nuclear medicine methods for various diseases have lost their relevance in clinical practice, there are still niche indications in which alternative methods cannot replace them. ^18^FDG-PET/CT combines metabolic and anatomical imaging and has proved sensitive in diagnosing chronic osteomyelitis and distinguishing septic from aseptic prosthetic loosening [39,40].

Until recently, ultrasonography was mainly used to guide joint aspiration and detect periarticular fluid collections and abscess formations. However, between 2005 and 2010, the use of contrast-enhanced ultrasound (CEUS) emerged as a valuable diagnostic modality in the evaluation of FRI. Intravenous administration of gas-filled microbubbles enables high-resolution, real-time tissue perfusion and vascularization assessment without exposure to ionizing radiation or nephrotoxic agents. The efficacy of CEUS has been demonstrated by significant perfusion disparities between aseptic and septic nonunions [41].

### 2.5. Emerging Technologies and Future Perspectives

Modern healthcare is typically characterized by step innovations rather than by leap or disruptive technologies. It is more likely that an informed and targeted combination of molecular, immunological, and computational methods will change our understanding of nonunions and FRI and improve care and outcomes.

In relation to the emerging field of artificial intelligence (AI), machine learning models are trained on extensive datasets, including imaging, laboratory, and clinical parameters. This training aims to predict infection risk, optimize diagnosis, and suggest diagnostic and therapeutic pathways [42]. Consalvo et al. developed an AI algorithm to identify imaging features in standard radiographs, distinguishing between osteomyelitis and Ewing sarcoma [43]. In addition, it is essential to recognize that artificial intelligence will also have a substantial impact on therapy optimization. Specifically, AI-powered decision support systems can optimize antibiotic use by recommending the most effective treatments based on patient data and local patterns of antibiotic resistance [44].

Alongside the development of artificial intelligence, the next generation of diagnostic tools is emerging. As mentioned above, a challenging diagnosis can be a low-grade infection that shows no clinical or radiological signs of infection. Therefore, the availability of a preoperative blood test to differentiate between aseptic conditions and low-grade infections would be of significant value. However, it is essential to remember that standard blood markers, such as white blood cell count or C-reactive protein, do not provide a definitive diagnosis. However, proteomics has the potential to provide valuable diagnostic biomarkers for differentiating septic from aseptic nonunion. In a preliminary analysis of plasma samples, Interleukin-6 (IL-6) and Hepatocyte Growth Factor (HGF) were found to be significantly higher in infected patients than in patients with healed and aseptic nonunion [45].

It is crucial to recognize that similar challenges in diagnosing nonunions and FRI are likely to persist for the next quarter century, even with the introduction of new technologies and methods, including standardization, regulatory approval, cost-effectiveness, and integration into clinical workflows.

## 3. Orthoplastic Management of Open Fractures and Fracture-Related Infections

The orthoplastic approach to open fractures and fracture-related infections combines principles from orthopedic and trauma as well as plastic and reconstructive surgery to control infection and achieve both skeletal stabilization and cutaneous reconstruction. This section summarizes current strategies, emphasizing that early, vascularized soft tissue coverage plays a pivotal role in facilitating healing and preventing persistent deep infection.

### 3.1. Epidemiology and Pathophysiology

The annual incidence of open fractures in industrialized countries is estimated at 11.5 per 100,000 people, with higher rates observed in cases of high-energy trauma [46]. The risk of infection depends on the severity of the soft tissue injury, neurovascular involvement, and both the timing and the adequacy of debridement. Consequently, reported infection rates range from 5% to over 30% for severe Gustilo-Anderson type IIIB and IIIC injuries [47,48].

The underlying pathophysiology involves direct bacterial contamination of the wound at the time of injury. This is aggravated by tissue ischemia, devitalization, periosteal stripping, and the presence of foreign material, which serves as a nidus for bacterial colonization. Therefore, managing these complex injuries requires meticulous debridement, stable bone fixation, reconstruction of neurovascular structures, and vascularized soft tissue coverage to establish a biological environment conducive to healing and less susceptible to (persistent) infection.

### 3.2. Principles of Orthoplastic Management

The core principles of orthoplastic management are adequate and repeated surgical debridement, stable skeletal fixation, early repair of neurovascular structures, and timely coverage with vascularized soft tissue, conjointly delivered by a multidisciplinary team.

In the context of orthoplastic reconstruction, meticulous surgical debridement is paramount and requires the excision of all non-viable tissue until resection margins of healthy, bleeding bone, muscle, and soft tissues are achieved. Techniques such as fluorescence-guided imaging and pulsatile lavage can aid in this process [49,50]. However, the role of high-pressure irrigation was questioned by the FLOW investigators, who favored low-pressure lavage [51]. Additionally, Hyperbaric oxygen therapy also seems to have a beneficial effect on wound healing in severe lower limb soft tissue injuries when implemented as an addition to standard trauma care [52]. A planned second-look operation is often mandatory to ensure the complete removal of any residual fibrin, contamination, or necrotic tissue. Once the wound is deemed macroscopically clean, the surgeon must select the optimal method for long-term mechanical stabilization, adapting the fixation strategy to the defect size and the condition of the soft-tissue envelope. External fixators provide versatile alignment with minimal additional trauma, whereas locked plates or intramedullary nails may be inserted immediately if adequate soft tissue coverage is achieved during the same operation. Robust evidence indicates that when performed after thorough debridement and under antibiotic coverage, immediate internal fixation (within 24–48 h) does not increase the risk of infection [53].

This approach has been termed the “fix and flap” concept, coined by Godina, which underscores the importance of early flap coverage, ideally within 72 h [54]. This principle is supported by data from various nationwide registries [53,54,55,56]. For proximal tibial defects, local muscle flaps like the gastrocnemius can be alternative solutions in low energy trauma scenarios. However, a significant proportion of open fractures result from higher-energy traumas and are accompanied by extensive soft-tissue injury [46,57]. In this so-called “zone of injury”, disruption of the subdermal plexus, impairment of angiosome-crossing connective vessels, and compromise of muscular microvasculature can render both local skin as well as muscle flaps considerably less reliable [58,59]. Therefore, free tissue transfer should be regarded as the preferred treatment choice in cases of complex extremity trauma in specialized centers with appropriate expertise and strong interdisciplinary collaboration [58]. As an alternative, synthetic dermal substitutes are an emerging trend for temporizing coverage before definitive reconstruction. However, recent studies have shown high failure rates in the presence of pre-existing infection, limiting their use to a reserve option when vascularized flaps are not feasible [60]. Therefore, in cases of established infections, a nuanced approach that carefully balances infection control with the timing of reconstruction is warranted.

### 3.3. Orthoplastic Surgical Strategies

Although it is beyond question that timely definitive reconstruction is associated with better outcomes, the optimal time window until definitive orthoplastic reconstruction in open extremity fractures cannot be precisely quantified at present due to insufficient data [61]. Likewise, evidence suggests that both the intervals from the initial injury and from the onset of infection are of limited relevance in the treatment of fracture-related infections [62,63]. Rather, a well-executed debridement should be of utmost priority in both scenarios.

Following adequate debridement, single-stage reconstruction combines definitive skeletal fixation and soft-tissue coverage within a single surgical session. The rationale for this approach is to minimize bacterial colonization by immediately covering exposed bone and hardware and thus to reduce the risk of infection. In addition, this approach can help reduce the need for repeated anesthesia, shorten hospital stay, and accelerate rehabilitation. Several studies have reported promising results, particularly lower deep infection rates, for single-stage reconstructions in carefully selected patients with open fractures, particularly those with a well-demarcated infection, good physiological reserve, and a suitable soft-tissue donor site [55,64,65]. However, a growing body of evidence suggests that rather than striving for the earliest possible definitive reconstruction, promptly achieving an adequate initial debridement is the most important predictor of success in the treatment of open fractures [66]. Particularly considering the advent of negative-pressure-wound-therapy, the ideal window until definitive reconstruction in open fractures may be extended up until about a week [67,68,69]. Rather, the necessity of multiple serial radical debridements until vital bone and soft tissue are achieved should determine the time interval until definitive reconstruction [70,71]. The same rule holds true for fracture-related infections, where the same rationale in favor of timely soft tissue coverage after debridement applies as a matter of principle [72,73]. Yet, just as the quality of initial debridement is crucial for open fractures, infection control is of paramount importance when reconstructing chronic osteomyelitis, and should thus dictate both the timing and staging of the respective treatment protocol.

In line with this, staged reconstruction divides the treatment process into distinct phases, according to the underlying therapeutic goals. Following radical debridement, it involves temporary skeletal stabilization and dead-space management, often using local antibiotic carriers (e.g., beads or spacers) to clear infection. Once the infection is controlled, definitive reconstruction is achieved, including final skeletal fixation combined with or without bone defect reconstruction. This approach allows for serial debridements and close monitoring to confirm infection clearance before committing to definitive skeletal reconstruction [72,73]. In this context, soft-tissue reconstruction with microsurgical free flaps is usually performed within the first phase, given the importance of well-vascularized tissue coverage for infection control, usually in the form of short interval staged approaches [72].

### 3.4. Types of Soft-Tissue Flaps for Coverage in Open Fractures and Fracture-Related Infections:

Despite their historical importance, local flaps now play a subordinate role, with exceptions for pedicled hand and finger flaps, and, in select cases of lower-energy injuries, robustly vascularized lower extremity muscle flaps like the gastrocnemius for the proximal and the soleus for the middle lower leg as well as the peroneus brevis for the lateral malleolus [74]. In cases of higher-energy trauma or chronic loco-regional infection, however, extensive inflammation and chronic ischemia in combination with advanced scarring and fibrosis, render local options less reliable [59,75]. Therefore, free flaps are the standard choice for soft tissue reconstruction in open fractures, particularly after high-energy trauma, and chronic osteomyelitis, as they tend to be safe and more reliable than local procedures in experienced hands.

When choosing a type of free flap, a distinction must be made between muscle or myocutaneous and fasciocutaneous or adipocutaneous flaps. While some studies showed higher partial necrosis rates for muscle free flaps in trauma-related reconstructions, presumably due to significantly larger flap sizes and higher metabolic demand, this does not seem to impact long-term outcomes, particularly regarding infection control in osteomyelitis cases [76,77]. However, cutaneous flaps offer other advantages, which is why they are increasingly used today. For one, they can achieve valuable protective sensibility as neurotized flaps through direct sensory coaptation, which is particularly advantageous for weight-bearing plantar defects. Furthermore, muscle flaps tend to form adhesions, whereas cutaneous flaps remain more pliable and are easier to elevate again for secondary osteosynthesis, which is of particular importance in staged reconstructions [71].

In summary, the selection of the appropriate flap for traumatic or infectious defects represents a multifactorial decision that must account for defect size and location, infection burden, patient comorbidities, quality of local tissues and the associated donor-site morbidity. While successful free-tissue transfer is dependent on significant microsurgical expertise, it should be regarded as the preferred standard of care interdisciplinary orthoplastic management of open fractures and fracture-related infections.

**Figure 3:**
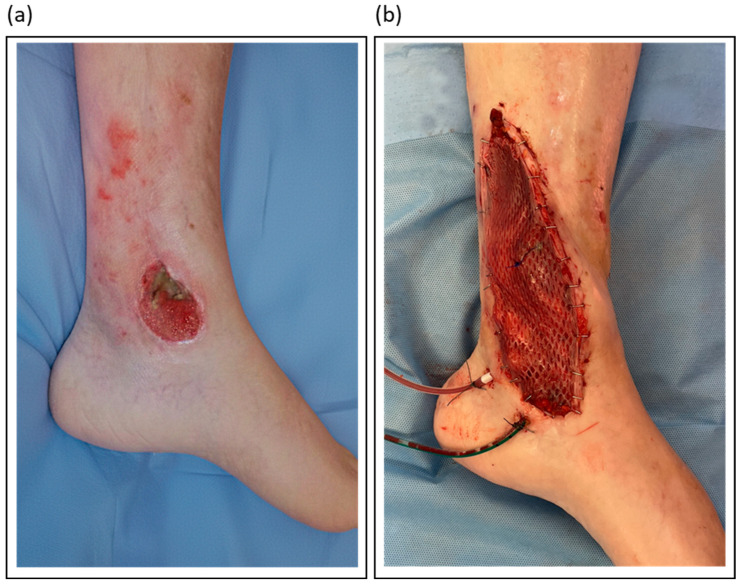
Illustrative orthoplastic management of a soft tissue defect following a distal lower leg fracture: (**a**) Clinical appearance of the chronic defect after initial debridement; (**b**) Intraoperative view of the same site after reconstruction with a gracilis free flap.

Table 3 outlines the author’s preferred flap types for various lower limb defect locations. Figure 3 illustrates a case of soft tissue reconstruction with a free flap.

### 3.5. Conclusion

In conclusion, the orthoplastic management of open fractures and fracture-related infections requires a dedicated multidisciplinary effort, considerable expertise, and ample resources. It is based upon the core principles of radical debridement and infection control, stable skeletal fixation, and robust coverage with well-vascularized soft tissue. While single-stage reconstruction can offer benefits in select cases, the staged approach remains the mainstay for complex infections, guaranteeing the inherent flexibility needed for effective surgical infection control.

Tailoring the surgical strategy to the individual patient, the specific wound environment, and the available institutional resources is critical to achieve the best possible outcome. Current evidence suggests that a dedicated, interdisciplinary orthoplastic approach can halve the rate of infectious nonunion and osteomyelitis in open fractures and double the rate of long-term infection control and limb salvage in fracture-related infections [79,80,81].

## 4. Antibiotic-Loaded Bone Substitutes in FRI and Nonunions

Radical debridement, essential for infection control and removal of necrotic tissue, frequently results in bone defects that require subsequent reconstruction to achieve successful healing [82]. Traditional treatment methods, such as autologous bone grafting combined with systemic antibiotics, remain the pillars of care. However, donor-site morbidity and limited graft availability have led to the development of various allogeneic and synthetic (alloplastic) bone graft alternatives over recent decades [83,84]. These “alloplasts” combine void filling with local antimicrobial efficacy [60], providing promising solutions to the persistent challenges posed by infected nonunions and FRI. An ideal bone graft should exhibit osteoinductive, osteoconductive, and antiinfective properties. Osteoinduction refers to the capacity to stimulate bone formation, whereas osteoconductivity enables bone ingrowth into the graft.

Synthetic bone graft substitutes, comprising ceramics, polymers, or composites, are engineered to mimic the structural and chemical properties of native bone. They offer advantages such as unlimited availability and reduced risk of disease transmission. Certain synthetic graft materials can be directly impregnated with antibiotics, delivering therapeutic benefits over traditional systemic antibiotic therapy or non-antibiotic-loaded bone grafts by providing higher local antibiotic concentrations and minimal systemic side effects [85]. Table 4 summarizes commercially available bone graft substitutes capable of antibiotic loading.

High local antibiotic concentrations established through antibiotic-loaded bone grafts can directly target pathogens in devitalized bone and biofilms, areas often unreachable by systemic antibiotics [2,13,87,88]. Local concentrations up to 100 times higher than systemic levels have been reported, which may explain their antimicrobial activity despite in vitro resistance findings, a crucial advantage when confronting the growing threat posed by multi-drug resistant pathogens [85]. Therefore, a synergistic strategy is employed where systemic antibiotics control widespread infection while local delivery eradicates pathogens in the poorly vascularized surgical site. While the duration of systemic therapy typically ranges from 6 to 12 weeks, the optimal length remains a subject of ongoing debate [89,90]. Furthermore, unlike polymethylmethacrylate (PMMA) beads, which require a second removal procedure [91], modern calcium sulphate-based or composite bone grafts are fully resorbable. This feature eliminates the need for additional surgery [2,92,93]. Regarding antibiotic release kinetics, PMMA is also suboptimal, typically releasing <10% of the loaded antibiotic [94,95]. In contrast, calcium sulphate-based substitutes release virtually all of the loaded antibiotic during dissolution [95].

### 4.1. Ceramic Bone Grafts

**Figure 4:**
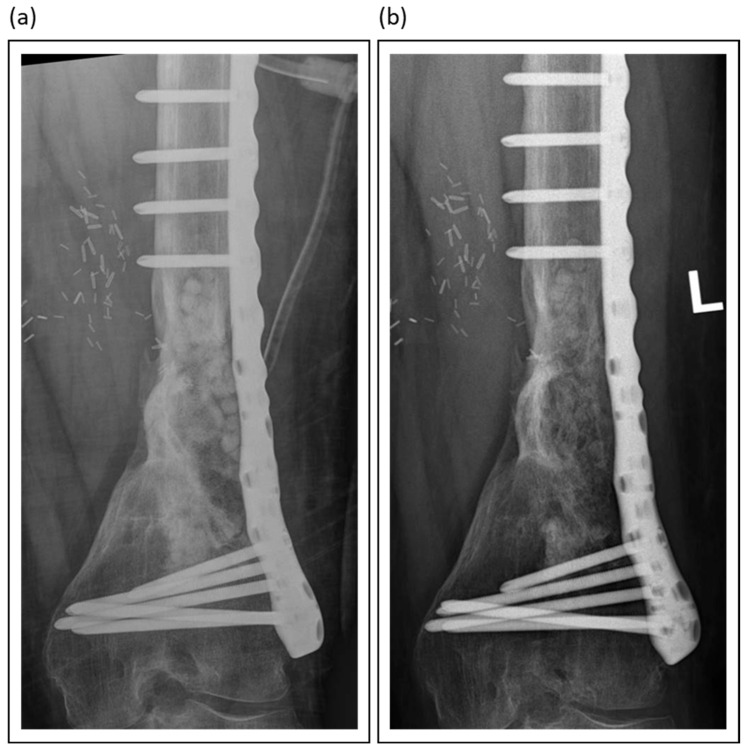
PerOssal^®^ as part of the treatment of chronic osteomyelitis in a distal femur: (**a**) Postoperative X-Ray image; (**b**) Follow-up X-Ray 2 years later. The PerOssal^®^-Beads are largely resorbed. No recurrence of infection was detected clinically.

Among synthetic grafts, ceramic-based materials, particularly those combining calcium sulphate with hydroxyapatite, such as PerOssal^®^ or Cerament^®^, have demonstrated promising outcomes. The inclusion of hydroxyapatite mitigates the early resorption of calcium sulfate, enhances biocompatibility, and reduces inflammatory responses, all while serving as a durable scaffold for bone regeneration [87,96]. Figure 4 presents an example of the use of absorbable PerOssal^®^ beads.

Comparisons among the various bone grafts are restricted by variations in study populations, defect sizes, and follow-up durations in the published literature. Few studies have evaluated multiple bone grafts within the same trial [97,98,99,100,101], and only a subset directly compares reinfection and revision rates of the respective bone grafts. To date, no randomized controlled trials exist, leaving surgeons reliant on cohort studies, some of which are conducted in close collaboration with manufacturers, raising concerns about potential conflicts of interest.

Cerament^®^ has one of the strongest evidence bases in the literature. It has demonstrated superb infection control and low complication rates in long bone nonunions and osteomyelitis [13,92,97,102] and has been extensively investigated in the context of diabetic foot infections [103,104,105,106,107,108,109,110,111,112,113,114]. Other bone grafts, like PerOssal^®^ [2,88,115] and Osteoset T^®^ [12,116,117,118] have also shown favorable reinfection rates and in some cases offer a broader antibiotic-loading capacity. Multiple systematic reviews have attempted to compare the divergent published cohort studies; however, each is constrained by the heterogeneity and limitations of the available data [82,87,96,119,120,121,122,123].

### 4.2. Potential Side Effects

Calcium sulfate-based beads offer effective antibiotic delivery but may resorb too quickly, sometimes leading to increased wound drainage and void-related complications. In contrast, composite grafts maintain structural support for a longer duration, thereby enhancing bone healing and reducing recurrence, as evidenced by lower infection and complication rates in comparative studies [97,99]. Their biphasic nature enables both short- and long-term resorption, promoting bone ingrowth while serving as a local antibiotic carrier.

One frequently reported complication is increased postoperative wound drainage (ranging from 5% to over 30% in tibial cases), though it is thought to be related to the hygroscopic nature of calcium sulfate. Notably, composite materials are associated with fewer drainage complications compared to pure calcium sulfate grafts [97,99,124]. However, most wound drainage episodes are described to be sterile and self-limiting, and rarely require surgical intervention [13]. Cerament^®^, an intraoperatively prepared antibiotic-loaded bone graft, is known to increase postoperative wound drainage [13]. Pre-hardened grafts like PerOssal^®^ may reduce such issues but no direct comparison of two composite materials in larger cohort studies is yet available.

Although only low systemic concentrations of antibiotics are generally observed when using antibiotic-loaded grafts, clinicians should still monitor and account for renal function. Both vancomycin and tobramycin are nephrotoxic agents primarily eliminated by the kidneys. In patients with pre-existing renal impairment, reduced clearance can lead to systemic accumulation and toxic trough levels, even when the antibiotics are delivered from local carriers. Therefore, a patient’s renal function should be carefully considered to avoid systemic toxicity when using these therapies [95,125,126].

### 4.3. Existing Gaps and Research Directions

Current concepts of alloplastic bone grafting have reached their limits in larger bone defects. The biological capacity appears constrained to minor bone defects of roughly 1–3 cm [127]. To overcome this challenge, current research focuses on incorporating osteoinductive proteins (e.g., bone morphogenetic protein 2 and parathyroid hormone) or bisphosphonates (e.g., zoledronic acid) into existing ceramic grafts [128,129]. While these adjuncts have shown promising effects in animal bone defect models, their efficacy in nonunion models [130] and in human patients remains to be determined.

Furthermore, research directions aim to manage complex orthopedic infections through multi-drug-loaded 3D-printed scaffolds that can be enhanced with 4D “shape-memory” properties for a superior anatomical fit [131]. In parallel, new long-acting antibiotics like oritavancin show significant promise for treating infections caused by challenging, multi-drug resistant pathogens [132].

### 4.4. Outlook

Over the last quarter century, antibiotic-loaded bone graft substitutes have revolutionized the management of FRI and nonunions. Their capacity to deliver high local antibiotic concentrations, potentially eliminate second-look surgeries, and confer osteoconductivity has made them invaluable in complex infection scenarios. Yet, clinical care and research stand at a pivotal juncture. Rigorous, large-scale head-to-head comparisons of the available grafts on the market are urgently needed to guide clinical decision-making and ensure patient safety.

Over the next 25 years, bone grafts will likely be integrated with biologically active adjuvants. Basic research will be crucial in identifying optimal combinations, paving the way for improved patient outcomes in the future.

## 5. Conclusions and Future Implications

The past quarter-century has witnessed profound transformations in the diagnosis and therapeutic management of nonunions and FRI. Diagnostic capabilities evolved from the foundational reliance on traditional microbiological cultures to the cutting-edge promise of metagenomic next-generation sequencing and artificial intelligence. Concurrently, therapeutic strategies have undergone substantial refinement, encompassing advancements such as orthoplastic management of infected open fractures and the local delivery of antibiotics via antibiotic-loaded bone substitutes.

The integration of these advancements necessitates highly specialized centers, which play an indispensable role in the most challenging cases, significantly impacting patient outcomes. This central role is increasingly vital given the global demographic shift towards an older population, which brings a higher burden of chronic diseases, immunosuppression, and prosthetic implants.

In conclusion, while significant progress has been made over the past 25 years, the journey is far from complete. The ongoing challenges, intensified by demographic shifts and antimicrobial resistance, underscore the necessity of an intelligent integration of advanced diagnostics, refined surgical techniques, innovative biomaterials such as multi-drug-loaded scaffolds. The field remains dynamic, demanding ongoing research, interdisciplinary collaboration, and a steadfast commitment to evidence-based practice to further enhance patient outcomes and continue to transform the management of nonunions and FRI for the next quarter-century and beyond.

## Figures and Tables

**Table 1:** Overview of biomarkers in the diagnosis of nonunions and fracture-related infections.

Biomarker	Type	Sensitivity [%]	Specificity [%]	Diagnostic Value	Comments
C-reactive protein (CRP) [26]	Acute-phase protein	62–100	64–96	Early reaction, beneficial for monitoring	Unspecific, elevated in many inflammatory conditions, widely used since the 1980s
White blood cell count (WBC) [21]	Cellular marker	21–42	89–94	Elevated in systemic responses	May be normal in chronic infections, basic test since the early 20th century
Procalcitonin (PCT) [27]	Calcitonin precursor	13–90	28–100	Distinguishes bacterial from viral infections	Often only mildly elevated in local infections, in clinical use since the early 2000s
Interleukin-6 (IL-6) [28]	Cytokine	47–100	53–95	Early marker of bacterial inflammation	Normalizes quickly with effective therapy, applied in diagnostics since mid-2000s
Alpha-defensin [23]	Antimicrobial peptide	95–100	95–100	Highly specific in periprosthetic joint infections	Typically tested in synovial fluid, expensive, introduced in orthopedics around 2014
Lipocalin-2 [29]	Neutrophil- associated protein	70–85	60–80	Promising for detecting bacterial inflammation	Mostly experimental since 2015
D-dimer [28]	Fibrin degradation product	60–96	32–93	May indicate infectious processes	Nonspecific, elevated in many non-infectious conditions, used in infection diagnostics since 2016

**Table 2:** Comparison of imaging modalities for nonunions and fracture-related infections.

Imaging Modality	CEUS	MRI	CT	FDG-PET/CT
Ionizing radiation	no	no	yes	yes
Perfusion assessment	yes	yes	no	yes
Bone assessment	indirect	yes	yes	yes
Availability	high	moderate	high	low
Portability	yes	no	no	no

**Table 3:** Flap types for various lower limb defect locations.

Location	Local Flap (When Indicated)	Free Flap (Preferred) *
Thigh	Tensor fasciae latae, Biceps femoris	ALT ± Vastus lateralis, PSC, Latissimus dorsi
Knee	Medial/Lateral Gastrocnemius	ALT ± Vastus lateralis, PSC, Latissimus dorsi
Proximal Lower Leg	Medial/Lateral Gastrocnemius	ALT, PSC, TDAP, Latissimus dorsi
Middle Lower Leg	Proximally based Soleus	ALT, PSC, TDAP, Latissimus dorsi
Distal Lower Leg	Distally based Soleus	ALT, MSAP/SCIP/TDAP, Gracilis
Lateral Malleolus	Distally based Peroneus brevis	ALT, MSAP/SCIP/TDAP, Gracilis
Foot	–	ALT, MSAP/SCIP/TDAP, Gracilis

* When choosing free flaps, cutaneous flaps are preferred, such as the anterolateral thigh perforator (ALT), medial sural artery perforator (MSAP), superficial circumflex iliac artery perforator (SCIP), thoracodorsal artery perforator (TDAP), or parascapular flaps (PSC) [78].

**Table 4:** Commercially available antibiotic-loaded bone graft substitutes. Herafil G (Heraeus) was excluded due to its discontinuation in recent years. Biopex^®^-R (HOYA) was excluded due to the absence of significant studies, aside from small case reports at the time of publication.

Product Name (Manufacturer)	Form	Composition	Antibiotic Loading
Cerament^®^ (Bonesupport)	Paste	60% calcium sulphate 40% hydroxyapatite	Gentamicin Vancomycin
Osteoset T^®^ (Stryker)	Beads	Calcium sulphate alpha-hemihydrate	Tobramycin
PerOssal^®^ (Osartis)	Beads	48.5% calcium sulphate 51.5% nanocrystalline hydroxyapatite	Vancomycin Gentamicin Tobramycin Rifampicin
Stimulan^®^ (Biocomposites)	Beads/Paste	Calcium sulphate hemihydrate	Vancomycin Gentamicin Tobramycin
Cerasorb^®^ (Curasan)	Beads/Paste	Beta-Tricalcium phosphate (β-TCP)	Vancomycin Gentamicin Meropenem [86]

## Data Availability

No new data were created or analyzed in this study. Data sharing is not applicable to this article.
